# Mimicking Pavlovian Conditioning with WSe_2_ Phototransistors

**DOI:** 10.1021/acsami.6c07765

**Published:** 2026-07-03

**Authors:** Andrea Sessa, Adolfo Mazzotti, Kimberly Intonti, Aniello Pelella, Loredana Viscardi, Nadia Martucciello, Stephen O’Sullivan, Vilas Patil, Paul K. Hurley, Lida Ansari, Farzan Gity, Antonio Di Bartolomeo

**Affiliations:** † Department of Physics “E. R. Caianiello”, 19028 University of Salerno, via Giovanni Paolo II, Fisciano, Salerno 84084, Italy; ‡ 9327 CNR-SPIN Salerno, via Giovanni Paolo II, Fisciano, Salerno 84084, Italy; § Tyndall National Institute, 261183 University College Cork, Lee Maltings, Dyke Parade, Cork T12 R5CP, Ireland; ∥ School of Chemistry, University College Cork, Cork T12 R5CP, Ireland

**Keywords:** WSe_2_, persistent photocurrent, neuromorphic, plasticity, associative learning, neural network

## Abstract

Neuromorphic engineering aims to bypass the energy and latency limitations of traditional Von Neumann architectures by emulating the biological efficiency of the mammalian brain. Two-dimensional materials, with their exceptional light-matter interaction, have emerged as prime candidates for next-generation optoelectronic artificial synapses. In this work, we demonstrate a robust optoelectronic synaptic device based on a mechanically exfoliated tungsten diselenide (WSe_2_) field-effect transistor. Unlike complex heterostructures, the proposed device exploits intrinsic defect-mediated charge trapping mechanisms to achieve neuromorphic functionalities. We report a reversible modulation of channel conductance, where optical stimuli induce potentiation via persistent photoconductivity and electrical gate pulses trigger depression. A key finding is the ability to selectively switch between Short-Term and Long-Term Plasticity simply by tuning the drain bias polarity, utilizing the asymmetry of Schottky contacts. To validate the device’s potential for bioinspired computing, we successfully emulate Pavlovian associative learning at the hardware level. These results establish exfoliated WSe_2_ as a simple yet versatile platform for light-stimulated neuromorphic computing.

## Graphical abstract

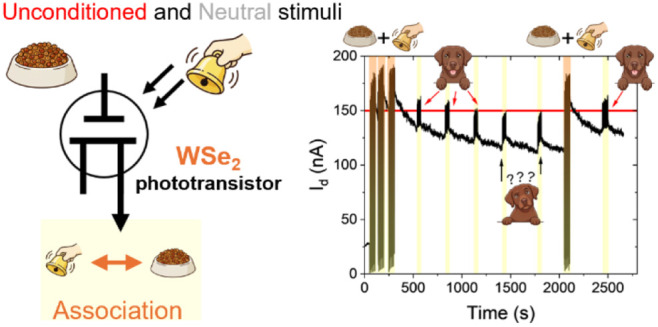

## 1 Introduction

The term *neuromorphic* was first introduced by Carver Mead in 1990.^1^ His core premise was simple: complex digital problems can be simplified by emulating the efficiency of the mammalian brain. Since then, research has advanced in two distinct directions: Artificial Neural Networks (ANNs) and biologically inspired hardware (Natural Neural Networks, or NNNs). ANNs currently form the backbone of machine learning and big data classification. However, they require intensive digital calculations, typically handled by central or graphics processing units. Conversely, neuromorphic engineering aims to overcome the limitations of the conventional Von Neumann architecture by building analog circuits that mimic biological principles. To achieve this, it is fundamental to replicate the connection between neurons, known as synapses. While silicon circuits have been developed to imitate neuron spiking and synaptic connections,^2,3^ achieving true brain-like intelligence on a standard chip remains a challenge.^4^ Consequently, research has shifted toward 2D materials.^5–7^ Their unique properties, specifically their atomically thin, dangling-bond-free surfaces and strong light-matter interaction, make them ideal candidates for optoelectronic artificial synapses. Furthermore, the diverse bandgaps of 2D materials allow for broad light absorption (from UV to infrared). This enables multiwavelength modulation, paving the way for devices capable of performing both sensing and computation.^8^

Among the investigated materials, tungsten diselenide (WSe_2_) emerges as an ideal candidate. WSe_2_ is a group-VI transition metal dichalcogenide exhibiting ambipolar charge transport,^9^ a layer-dependent electronic band structure, with a direct bandgap in the monolayer limit and an indirect bandgap in few-layer and bulk forms,^10^ strong spin–orbit coupling with a large valence-band splitting (∼460 meV),^11^ high carrier mobility,^12^ and pronounced light–matter interaction characterized by strong excitonic effects and high optical absorption.^13^ In addition, WSe_2_ presents mechanical flexibility, environmental stability, and compatibility with scalable device fabrication.^14^ WSe_2_ has been extensively investigated for a broad range of applications, including field-effect transistors,^15–17^ field emission,^18^ photodetectors and optoelectronic devices,^19,20^ valleytronic and spintronic systems,^21^ nonvolatile memories,^22,23^ and neuromorphic devices.^24,25^

Numerous studies have demonstrated that charge trapping and detrapping processes associated with intrinsic defect states constitute the dominant mechanism enabling neuromorphic functionalities in WSe_2_-based devices.^26^ Importantly, these trap states originate from native structural defects, such as selenium or tellurium vacancies and grain boundaries, and therefore do not require additional material engineering or postprocessing treatments.^27^

Building on this understanding, recent progress in WSe_2_ optoelectronic neuromorphic devices has been driven by complex van der Waals heterostructures and floating gate architectures. Huang et al. demonstrated synaptic modulation in SnSe_2_/WSe_2_ heterojunctions from interfacial charge transfer.^24^ Similarly, Su et al. utilized floating-gate WSe_2_/MoS_2_ diodes for stable paired-pulse facilitation (PPF) and long retention.^28^ Zeng et al. employed a BP/PO_*x*_/WSe_2_ synaptic transistor for robust short- and long-term plasticity (STP and LTP) and neuromorphic visual processing.^29^ Graphene (Gr)-based heterostructures were reported by Kim et al. (WSe_2_/Gr) using asymmetric charge trapping for multilevel synaptic functions,^30^ while Tang et al. tuned potentiation and depression by light intensity for reconfigurable behavior.^31^

Beyond floating gates, heterostructures also enable synaptic functions. Wang et al. realized MoS_2_/WSe_2_ memtransistors via carrier separation.^32^ Ma et al. achieved ultralow-power STP-to-LTP transitions in Lewis acid-doped WSe_2_ through defect-assisted photogating.^33^ Near-infrared operation was achieved by Lu et al. using WSe_2_ with lanthanide-doped nanoparticles through frequency conversion.^34^ Finally, Luo et al. demonstrated a synaptic memristor based on layered WSe_2_ nanosheets, exhibiting both STP and LTP through trap-controlled charge transport.^35^

In this work, we present a systematic investigation of the photoresponse in FETs based on exfoliated WSe_2_ flakes. The device features asymmetric Schottky contacts characterized by significant disparities in their respective contact areas. Comprehensive electrical characterization under both dark and illuminated conditions reveals a controllable PPC. Specifically, the transistor exhibits a transition between STP and LTP that is selectively governed by the polarity of the applied drain bias.

This mechanism enables straightforward control over the synaptic response solely through engineered contact asymmetry, a configuration that is easily achievable via various fabrication strategies, such as top-and-bottom contact geometries,^36^ modulation of electrode^37^ or flake thickness, or by using different contact metals.^38^ The inherent simplicity of this architecture suggests high generalizability across other material systems. Furthermore, by exploiting the interplay between asymmetric Schottky barriers and trap-mediated photogating, we experimentally demonstrate associative learning at the device level, analogous to classical Pavlovian conditioning. Collectively, these results highlight the suitability of WSe_2_-based optoelectronic devices for next-generation neuromorphic computing and bioinspired learning architectures.

## 2 Materials and Methods

WSe_2_ flakes, characterized by the atomic structure shown in Figure 1a,^39^ were mechanically exfoliated and transferred onto an 85 nm SiO_2_/Si substrate. Ni/Au (20 nm/200 nm) contacts were fabricated via metal evaporation following standard photolithography and liftoff steps, resulting in the device displayed in Figure 1b. Specifically, the device features highly asymmetric Ni/Au electrodes with respect to their interfacial area with the WSe_2_ flake. Designating these terminals as Contact A and Contact B, their respective contact areas are approximately 138 μm^2^ and 5 μm^2^. Material quality was verified by Raman spectroscopy (Figure 1c), showing the spectrum of the multilayer WSe_2_ flake exhibits well-separated peaks corresponding to the *E* ^1^_2g_/*A*_1g_ and 2LA­(M) modes. As previously noted, such multilayer flakes are selected to ensure higher carrier concentrations.^40^

**Figure 1:**
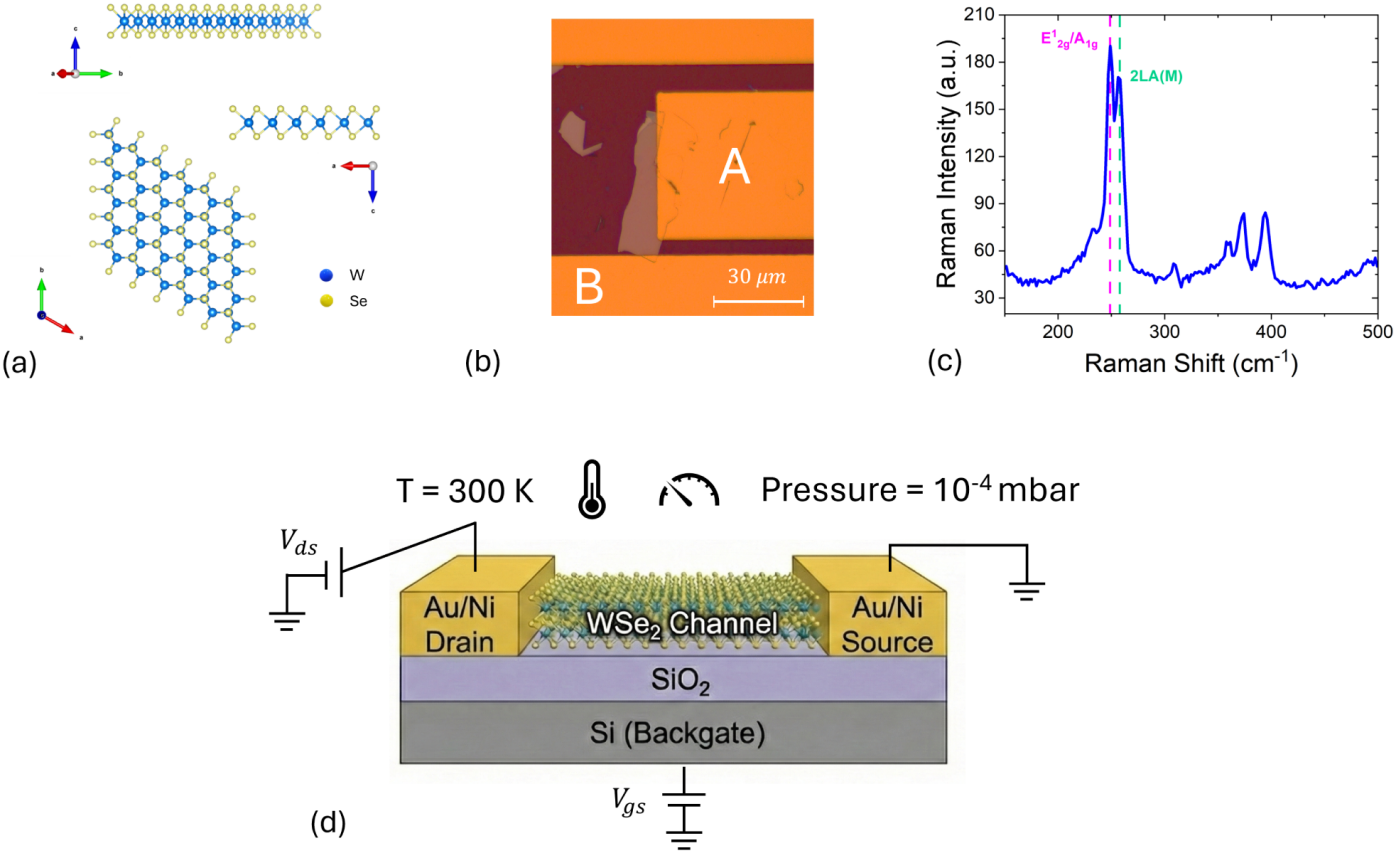
Exfoliated WSe_2_-based field-effect transistor. **a.** Atomic structure of WSe_2_. **b.** Optical image of the flake with asymmetric Ni/Au contacts. **c.** Raman spectrum of the multilayer WSe_2_ flake. **d.** Schematic of the device with measurement setup and conditions.

The measurement setup and device schematic are illustrated in Figure 1d. Electrical characterization was performed in a two-probe configuration using nanoprobes, with the back-gate voltage applied directly through the sample holder. Data were acquired using a Keithley 4200 Semiconductor Characterization System at room temperature under vacuum (10^–4^ mbar). Measurements were conducted under high vacuum to isolate the intrinsic device physics from the influence of atmospheric adsorbates, which are known to modulate the transport and photoconductive properties of TMDs.^41^ Despite this choice, the device maintains robust electrical and synaptic functionality under ambient conditions, as demonstrated by the characterization provided in Figure S2 of the Supporting Information. All the measurements presented in this work were conducted on a single representative device, as shown in Figure 1a. Finally, the device photoresponse was analyzed using white light from a supercontinuum laser source, delivering white light in the spectral range from 420 to 2400 nm with a maximum optical power of 16.5 mW.

## 3 Results and Discussions

### 3.1 Persistence of the Photocurrent

The electrical transport properties of the device were probed by measuring the drain-source current (*I*_d_) as a function of the drain-source voltage (*V*_ds_, output characteristics) and gate-source voltage (*V*_gs_, transfer characteristics). Figure 2a (black line) displays the current–voltage (IV) characteristics acquired in the dark with a grounded gate. The curve exhibits a pronounced difference between positive and negative drain bias polarities, characteristic of an asymmetric back-to-back Schottky barrier structure.^42^ The reverse leakage current in a Schottky diode is governed by thermionic emission and described by the following formula^43^

1 $$I=SA^{*}T^{2}e^{-\frac{q\phi_{B}}{kT}}$$

where *S* represents the effective contact area, A* is the effective Richardson constant, and the exponential term relates to the Schottky barrier height *q*ϕ_*B*_. The Schottky barrier heights at both contact interfaces were evaluated by performing temperature-dependent output characterization at a grounded gate from 300 to 380 K (see Figure S3a in the Supporting Information). By applying the standard Richardson plot analysis (Figure S3b), we extracted nearly identical barrier heights of 0.48 ± 0.02 eV for contact A (probed at positive *V*_ds_) and 0.53 ± 0.03 eV for contact B (probed at negative *V*_ds_). A comprehensive discussion of the Richardson analysis can be found in Section 3 of the Supporting Information.

**Figure 2:**
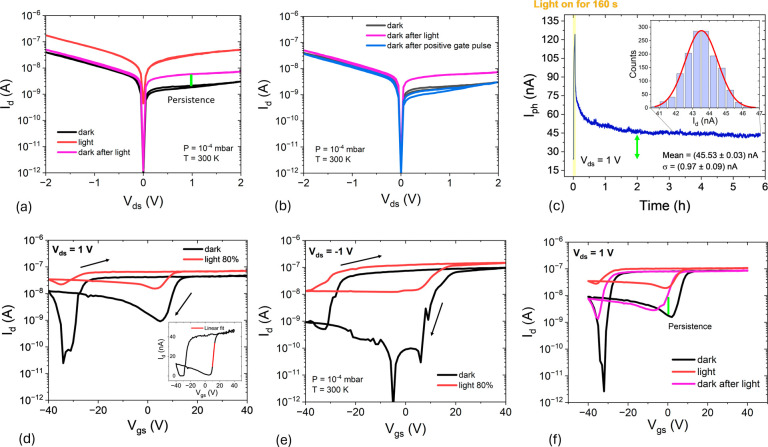
Optoelectronic characterization and persistent photoconductivity of the WSe_2_ FET. **a.** Output characteristics measured in the dark state, under illumination, and immediately after switching off the light. **b.** Effect of a gate pulse on the device recovery. Current–voltage curves show the persistent photocurrent and the restoration of the initial state after a positive gate pulse of 5 V for 20 s. **c.** Time-resolved photoresponse illustrating the persistence effect. The light is turned on for 160 s, followed by 6 h of data collection in the dark. The inset displays a statistical analysis of the current stability. **d, e** **.** Transfer characteristics comparing dark and illuminated states at a drain voltage of 1 V (d) and −1 V (e). The black arrows indicate the gate sweep direction. **f.** Evolution of the transfer curves at a drain voltage of 1 V acquired before, during, and after light exposure.

To ensure that the observed current asymmetry is not an artifact of potential barrier inhomogeneities, we further conducted a Werner-Güttler analysis (Figure S3c).^44^ This analysis revealed a standard deviation from the mean barrier height of approximately 10^–2^ eV for both interfaces. This negligible value falls within the experimental uncertainty associated with the Richardson method, confirming that the contact behavior is not significantly influenced by barrier fluctuations. Consequently, these findings provide strong evidence that the electrical asymmetry of the device is fundamentally rooted in the geometric disparity of the contact areas.

The device was stimulated by the light from a supercontinuum laser of nominal power *P*_nom_ = 16.5 mW. The incident optical power density was calculated by normalizing the active channel area *S*_active_ = 33.25 μm^2^ to the laser spot size *S*_spot_ = 1 mm^2^ according to

2 $$P_{\mathrm{inc}}=\frac{P_{\mathrm{nom}}\cdot S_{\mathrm{active}}}{S_{\mathrm{spot}}}$$

Unless otherwise specified, measurements were conducted at 80% of the maximum laser power, corresponding to an incident power of *P*_inc_ (80%) = 0.44 μW. Upon illumination, photoexcitation and photogeneration of electron–hole pairs increase the free carrier density, leading to the symmetrization of the IV curve observed in Figure 2a (red line).^45^ After switching off the light, the IV curve (Figure 2a, purple line) deviates from the initial dark state. Specifically, at positive *V*_ds_, the current fails to immediately relax to the preillumination level. This residual current, indicated by the green line in Figure 2a, is attributed to persistent photoconductivity.^46^ The absence of PPC at negative *V*_ds_ underscores the critical role of contact asymmetry in this mechanism. Importantly, applying a positive gate pulse (*V*_gs_ = 5 V) of 20 s suppresses the PPC, restoring the device to its pristine dark state (Figure 2b, blue line).

To elucidate the relaxation dynamics and the magnitude of the persistent current, time-dependent drain current measurements were performed at a grounded gate with a fixed drain bias of (*V*_ds_ = 1 V). Following a 160 s optical pulse, the device does not relax immediately to the initial dark state but settles into a metastable, high-conductivity level. As shown in Figure 2c, the PPC, highlighted by the green arrow, remains stable for over 6 h. The stability of this excited state during the final 3 h of measurement is detailed in the inset of Figure 2c; the recorded current values exhibit a Gaussian distribution with a mean value of 45.53 nA and a standard deviation of 0.97 nA. This exceptional stability suggests a fundamental alteration in the conduction state, which can be reset via gate bias.

The transfer curve was investigated by sweeping *V*_gs_ from 40 V to −40 V under both dark and illuminated conditions, at *V*_ds_ = 1 V (Figure 2d) and *V*_ds_ = −1 V (Figure 2e). As reported in Figure S1 of the Supporting Information, the gate leakage current was thoroughly monitored. The device displays ambipolar transport behavior, consistent with literature reports for similar WSe_2_ devices.^47,48^ Notably, the charge neutrality point (Dirac point) is shifted toward positive *V*_gs_ values, indicating p-type doping. In nickel-contacted WSe_2_, p-doping is typically attributed to tungsten vacancies, which represent the dominant defect in such material.^49^ Furthermore, a pronounced hysteresis is observed in the transfer characteristics under both positive and negative V_ds_ biases, driven by the forward and reverse gate voltage sweeps. In TMD-based field-effect transistors, this hysteretic behavior is generally attributed to charge trapping and detrapping mechanisms occurring in intrinsic traps, at the oxide-semiconductor interface and within the dielectric layer, which collectively screen the applied gate field during the voltage sweeps.^41,50^

The signature of persistent photoconductivity is also evident in the transfer characteristics displayed in Figure 2f. Immediately following illumination, the transfer curve retains a rigid shift toward negative gate voltages, pointing to positive charge storage.^50^ Even after the light source is extinguished, this shift persists; consequently, the current at zero gate bias remains elevated compared to the preillumination level, confirming the presence of the PPC effect (indicated by the green line).

To elucidate the photoconduction mechanisms governing the device operation, a power-dependent analysis was conducted. The temporal evolution of the drain current was recorded at a fixed drain bias and *V*_gs_ = 0 V. Optical excitation started at *t* = 100 s, with the incident laser intensity varying from 10% to 80% of the maximum power. The photocurrent (*I*_ph_) was extracted as the differential current between the illuminated and the dark level

3 $$I_{\mathrm{ph}}=I_{\mathrm{light}}-I_{\mathrm{dark}}$$

*I*_ph_ is plotted versus *P*_inc_ in a log–log scale in the inset of Figure 3a. According to the standard power-law dependence,

4 $$I_{ph}=AP^{\alpha }$$

the exponent α provides insight into the generation/recombination kinetics and charge collection efficiency.^51^ A fit to the experimental data, reported in the inset of Figure 3a, yields α ≈ 0.6, indicating a sublinear photoresponse. Values of α in the range 0.5 < α < 1 are typically indicative of one specific trap-limited process, where the collection efficiency of photogenerated carriers is hindered by the presence of trapping intragap states.^52^

**Figure 3:**
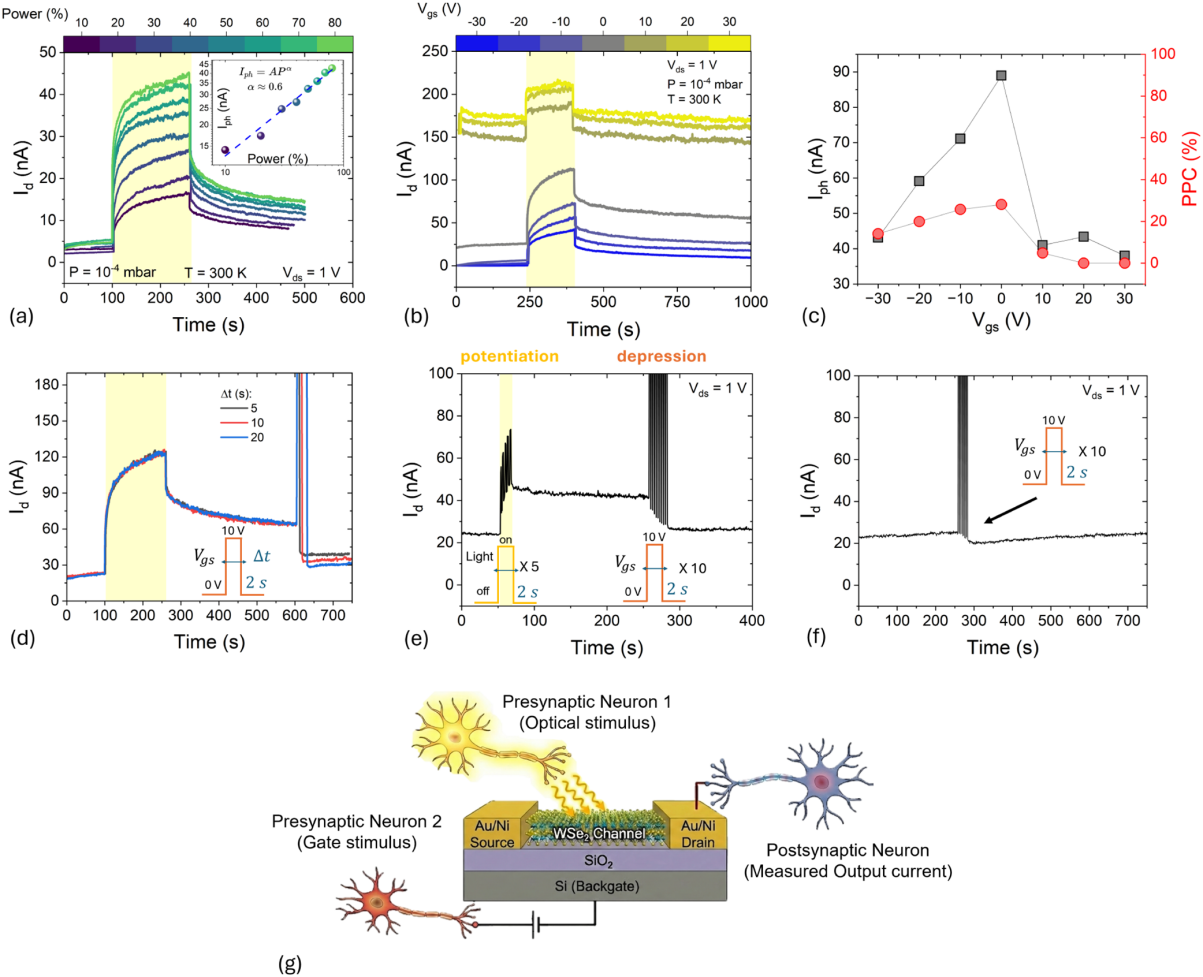
Power and gate modulation of the WSe_2_ optoelectronic synapse. **a.** Power-dependent photoconductivity. The inset shows the log–log plot of photocurrent versus optical power, revealing a sublinear behavior with a scaling exponent of 0.6. **b.** Time-resolved current response measured at a fixed drain bias while varying the gate voltage from −30 to 30 V in 10 V increments. **c.** Dependence of the photocurrent and persistent photocurrent on the applied gate voltage. **d.** Erasure of the persistent photoconductivity using positive gate voltage pulses of varying durations. **e, f.** Control measurements demonstrate that positive gate pulses effectively reset the persistent state (e) but induce no change when applied to the initial dark state (f). **g.** Schematic illustration of the costimulated synaptic device architecture based on a WSe_2_ transistor.

Furthermore, the dependence of the photoconductivity on the gate bias was investigated to study the interplay between electrostatic doping and carrier trapping. The time-resolved current response was measured while stepping *V*_gs_ from −30 V to +30 V in 10 V increments, as displayed in Figure 3b. The data reveal different behavior between the negative and positive gate bias regimes. At *V_gs_* = 0 V, the device exhibits a slow temporal rise upon illumination and a significant PPC following the extinction of the light source. This slow dynamic response is less pronounced at negative gate voltages. Conversely, at positive gate voltages, the photoresponse mechanism is fundamentally different: the device shows rapid switching dynamics with no observable persistence. This confirms the observations in Figure 2b, where a positive gate bias was shown to effectively quench the PPC. Figure 3c summarizes the gate dependence of the photocurrent magnitude (black squares) and the PPC ratio (red dots). The PPC ratio is defined as the residual current after light extinction relative to the photoinduced current, described by

5 $$PPC\left(\%\right)=\frac{I_{\mathrm{dark},\mathrm{post}}-I_{\mathrm{dark},\mathrm{pre}}}{I_{\mathrm{light}}-I_{\mathrm{dark},\mathrm{pre}}}$$

This metric quantifies the fraction of current that persists. The maximum photocurrent and PPC occur at *V*_gs_ = 0 V. The PPC is effectively suppressed at positive gate biases and slightly reduced at negative biases. The correlation between the modulation of *I*_ph_ and the PPC suggests that the same physical mechanism governs both the steady-state photoresponse and the persistent trapping effects.

The possibility of electrically erasing the persistent photocurrent was systematically investigated by applying positive gate voltage pulses of controlled duration. Figure 3d illustrates the device response to varying the pulse width of the reset signal. It is observed that a positive *V*_gs_ pulse of at least 20 s is required to fully suppress persistence and restore the initial dark current level, attesting to the robustness and high stability of the photoexcited state. Furthermore, this dynamic response implies that the modulation signals need not be continuous; both the optical excitation (potentiation) and the electrical gate reset (depression) can be discretized into trains of shorter pulses. This capability allows for the fine-tuning of the device conductance state through the summation of discrete stimuli, a key feature for mimicking synaptic weight updates.^53^ A sequence of 5 optical pulses (period *T* = 2 s) was employed to progressively excite the current, establishing a distinct, elevated conductance state. This state can be subsequently erased by applying a corresponding train of 10 positive gate voltage pulses, effectively enabling a reversible write-erase protocol, as shown in Figure 3e. To verify that the posterasure baseline corresponds to the nonexcited dark level, the same positive gate sequence was applied to the device in the dark. The current exhibited only a minor transient decrease before fully relaxing to the initial starting level, confirming its stability (Figure 3f).

These transport characteristics establish a robust framework for implementing neuromorphic functionality, as conceptually illustrated in Figure 3g. The optical modulation of the drain current mimics the excitatory postsynaptic current (EPSC) observed in biological synapses. In our device, the photogeneration of electron–hole pairs enhances the channel conductivity, emulating this excitatory response. Crucially, efficient neural processing also requires a mechanism for depotentiation, i.e., the ability to forget obsolete information.^54^ The net synaptic weight is determined by the summation of these excitatory and inhibitory signals. We emulate this integration by utilizing light pulses for potentiation and positive gate voltage for depression. Consequently, the device operates as an elementary neural node with two distinct inputs (optical and electrical) and a single output (drain current).^55^

While the gate-based control aligns with conventional three-terminal synaptic transistors,^56^ the optical input offers a distinct advantage: the highly stable and tunable PPC allows for the precise emulation of synaptic plasticity. Synaptic plasticity is widely recognized as the primary biological mechanism for information storage, serving as the bridge between transient sensory inputs and the formation of permanent memories. In our device, this phenomenon is emulated by encoding optical stimuli into a long-term potentiated state via PPC. Furthermore, the device provides the capability to inhibit or depress this state through the application of a gate voltage, thereby replicating the dynamic modulation of synaptic weights.

To elucidate the origin of the persistent photocurrent, we propose a band-structure model based on defect-mediated charge dynamics, as schematically illustrated in Figure 4. In the construction of the schematic band diagram, the work functions for both Ni and WSe_2_ were assumed to be approximately 4.5 eV.^57^ The bandgap and electron affinity utilized were 1.1 and 3.7 eV, respectively.^58,59^ Intrinsic defects introduce acceptor-like states, deep within the bandgap, inducing WSe_2_ p-type doping.^60,61^ These defects capture electrons, thereby generating free holes in the valence band. Consequently, the equilibrium band alignment (Figure 4a) exhibits a barrier profile that favors hole injections.

**Figure 4:**
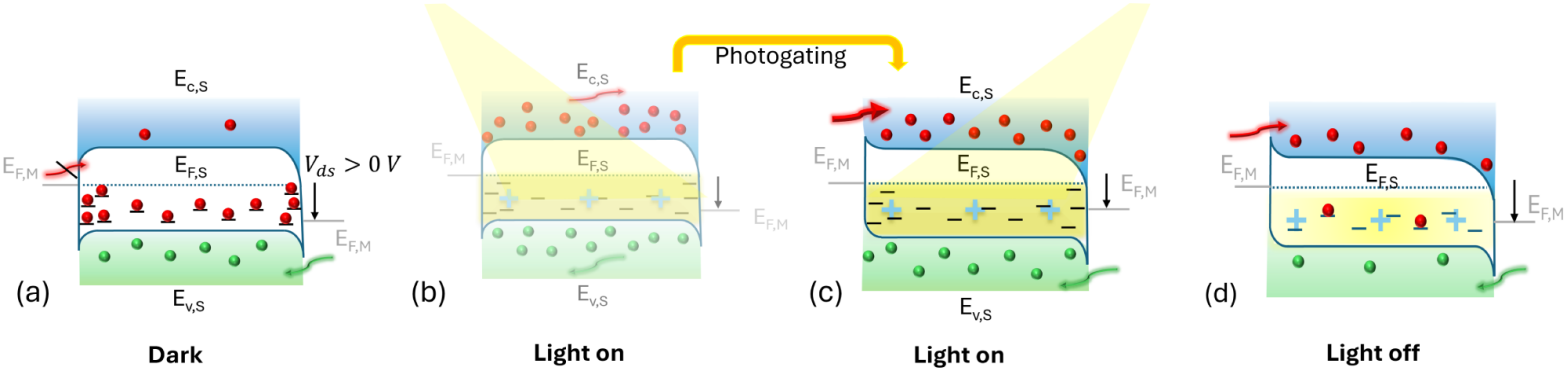
Proposed band-structure model illustrating the defect-mediated persistent photocurrent. **a.** Equilibrium energy band diagram in the dark state. Acceptor-like trap states within the bandgap result in p-type doping and the barrier profile favors hole injection. **b, c.** Incident photons ionize the trap states, generating free electrons and leaving localized positive charges. This creates a virtual positive gate effect (b) that induces downward band bending, lowering the Schottky barrier for electron injection and switching the device to an n-type conduction regime (c). **d.** The model also accounts for the persistent state caused by hindered carrier recombination.

Upon illumination, the incident photon energy ionizes these occupied trap states. The electrons are excited into the conduction band, while the positively charged vacancies remain localized. These fixed positive charges act as a virtual positive gate.^62^ This photogating effect induces a downward band bending, effectively shifting the device from a p-type to an n-type conduction regime (Figure 4b-c). This transition lowers the effective Schottky barrier for electron injections, resulting in the observed dominance of electron transport under illumination and the corresponding negative shift (left shift) of the transfer curve (red line in Figure 2d). The slow temporal rise of the photocurrent (Figure 3a) and its sublinear power dependence further corroborate a transport mechanism governed by the kinetics of traps. Following the extinction of the light source, the system does not immediately relax to equilibrium. The restoration of the dark state requires the recapture of electrons by the ionized vacancies. However, this recombination process is suppressed because the electric field rapidly sweeps electrons toward the drain, reducing the time available for recombination. This difficulty in reoccupying the trap states maintains the “virtual gate” effect, leading to a metastable band configuration where the transfer curve remains shifted and the current at *V*_gs_ = 0 V is higher, as confirmed by the postillumination transfer sweep in Figure 2f. This mechanism explains the remarkable long-term stability of the excited state reported in Figure 2c. This model also accounts for the gate-dependent photoresponse. When a large positive gate bias is applied, the channel is flooded with electrons (majority carriers). The high electron density significantly increases the capture probability, facilitating the rapid reoccupation of trap states upon light extinction and effectively suppressing the PPC. Conversely, at negative gate biases, the gate electrostatic field counteracts the local photogating, thereby reducing both the net photocurrent magnitude and the PPC, consistent with the data in Figure 3c.

### 3.2 Light-Stimulated Neuromorphic Behavior

To evaluate the device’s capability to emulate biological temporal processing, we characterized the drain current response under pulsed optical stimulation varying timing parameters. Figure 5a displays the time-resolved drain current at positive drain bias (*V*_ds_ > 0) stimulated by a train of 10 short optical pulses with variable interspike intervals (*t*_off_), ranging from 50 to 800 ms. This measurement protocol permits the estimation of Paired-Pulse Facilitation (PPF), a fundamental form of synaptic plasticity observed in biological neural networks. In biological systems, PPF manifests as an enhancement of the excitatory postsynaptic current when a second spike closely follows a preceding one. To quantify this effect in our artificial synapse, the PPF index is defined as^63^

6 $$PPF\left(\%\right)=\frac{A_{2}}{A_{1}}\times 100\left(\%\right)$$

**Figure 5:**
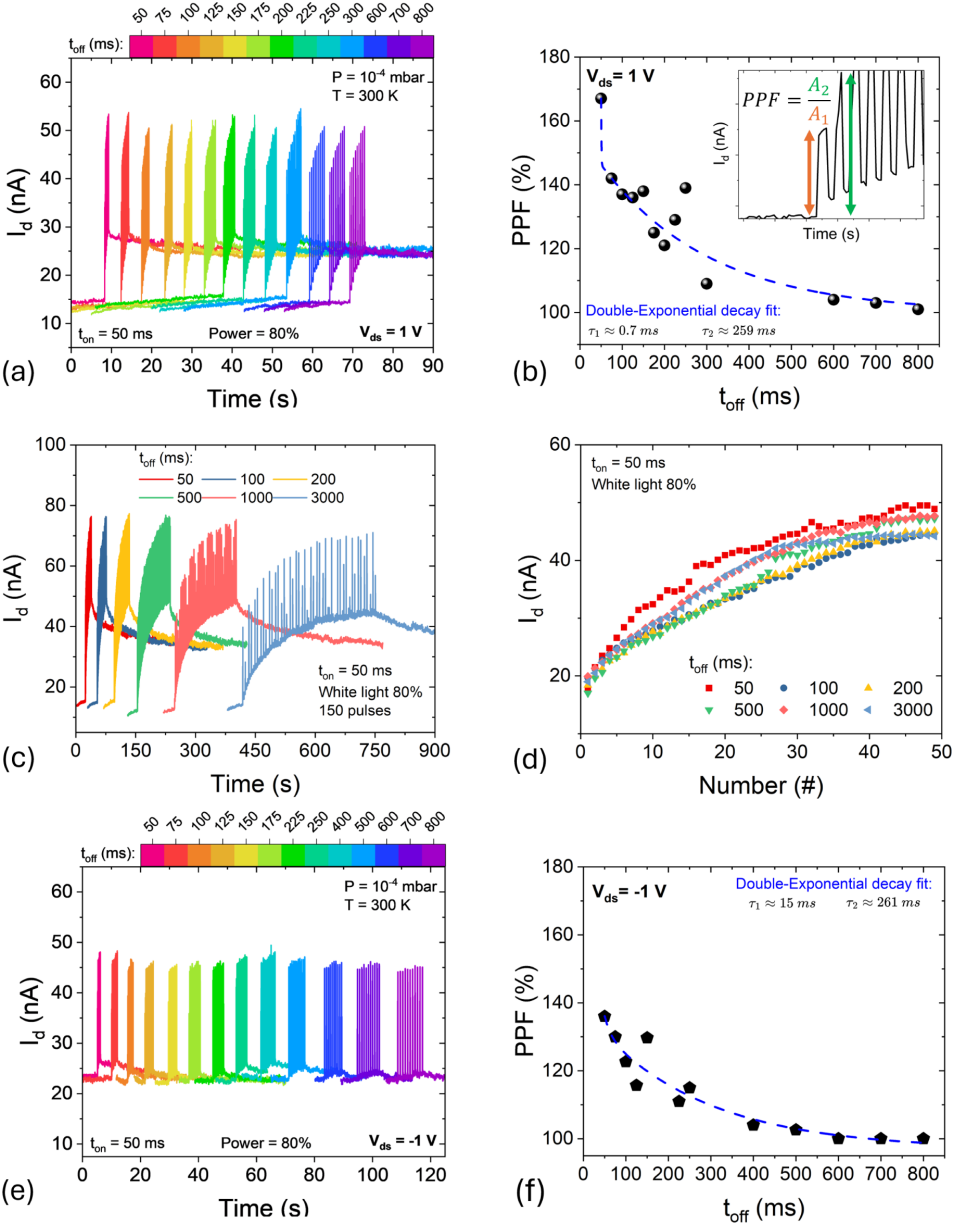
Device response to optical pulse stimulation at different drain voltages. **a.** Dynamic response to 10 consecutive light pulses with a fixed on-time of 50 ms and varying off-times, measured at *V*_ds_ = 1 V. **b.** PPF percentage plotted as a function of the time interval between pulses at a drain voltage of 1 V. The dashed blue line represents a double-exponential decay fit. **c.** Response to a train of 150 consecutive light pulses with varying off-times, recorded at a fixed drain voltage of 1 V. **d.** Current increment as a function of the number of pulses. The curve shows that saturation occurs after approximately 50 pulses. **e.** Dynamic response to 10 consecutive light pulses with a fixed on-time of 50 ms and varying off-times, measured at a negative drain voltage of −1 V. **f.** PPF percentage plotted as a function of the time interval between pulses at a drain voltage of −1 V, fitted with a double-exponential decay function.

where *A*_1_ and *A*_2_ represent the peak current amplitudes triggered by the first and second pulse, respectively (see the inset of Figure 5b). As shown in Figure 5b, the PPF index reaches its maximum of 167% at the shortest pulse intervals and decays asymptotically toward 100% (indicating no facilitation) as the interval increases. The temporal decay of the PPF is fitted using a double-exponential function, consistent with biological models:

7 $$PPF=C_{0}+C_{1}\exp\left(-\frac{\Delta t}{\tau_{1}}\right)+C_{2}\exp\left(-\frac{\Delta t}{\tau_{2}}\right)$$

where *C*_0_ represents the baseline (100%), while τ_1_ and τ_2_ denote the characteristic relaxation times for the rapid and slow phases, respectively. Notably, the extracted time constants are τ_2_ ≈ 1 ms and τ_2_ ≈ 100 ms. These values are significantly faster than those reported in many comparable 2D material-based devices and well align with the natural time scales of biological synapses (ms to s).^64,65^

To further investigate the device stability and response to sustained stimulation, we exposed it to a train of 150 optical pulses (Figure 5c). Figure 5d illustrates the drain current evolution as a function of pulse number until saturation. Both the figures reveal Spike-Rate Dependent Plasticity (SRDP): the cumulative increase in conductance is dependent on the frequency of the stimulus. As the time interval between consecutive pulses increases, the cumulative effect diminishes.

A contrast in synaptic behavior is observed when the polarity is reversed to negative drain bias. Due to the suppression of the PPC at *V*_ds_ = −1 V, the device exhibits negligible memory retention. Consequently, the current rapidly relaxes to the dark level upon light extinction, preventing the efficient summation of postsynaptic currents unless the pulse interval is extremely short. This results in a significantly reduced PPF index compared to the positive bias regime, e.g., 136% at minimal spacing, although the characteristic times remain comparable. This polarity-dependent behavior provides a versatile mechanism to switch the device functionality between Long-Term Potentiation (LTP) and Short-Term Potentiation (STP). From a neuromorphic engineering perspective, this duality is critical as it emulates the brain’s ability to optimize energy and processing resources by filtering irrelevant information (STP) while consolidating significant events into memory (LTP). In our architecture, this transition is controlled by switching from positive *V*_ds_ (LTP mode) to negative *V*_ds_ (STP mode). In particular, a double exponential fit of the photocurrent decay under both positive and negative *V*_ds_ polarities are provided in Figure S4 of the Supporting Information. This analysis confirms the distinct short- and long-term retention characteristics of the photocurrent at the two drain voltages, consistent with the observed bias-dependent synaptic behavior.

The difference in persistence between positive and negative bias regimes is a direct consequence of the geometric asymmetry of the Schottky contacts. Under a positive bias applied to Contact A, electrons are injected from the grounded, smaller-area Contact B. The injection current is directly proportional to the effective contact area. Consequently, this smaller electrode acts as a transport bottleneck, injecting a significantly lower density of electrons into the channel. Because the recombination rate is strictly dependent on the free-carrier concentration, this restricted electron density severely reduces the probability of electrons being captured by ionized trap states. This hindered recombination sustains the photogenerated “virtual gate” effect, ultimately leading to the observed persistent photocurrent. Conversely, under a negative bias, electrons are injected from the larger-area Contact A. This extended geometric area facilitates a substantially higher injection of charge carriers into the channel. The resulting high density of free electrons drastically increases their capture probability by the localized positive traps. This promotes rapid and efficient charge recombination, which quickly quenches the photogating effect and results in a fast relaxation of the device current.

This result highlights the potential of utilizing asymmetric Schottky barrier field-effect transistors to engineer controllable and reconfigurable synaptic plasticity for neuromorphic computing. Overall, the demonstrated synaptic behavior indicates that the optically driven dynamics, retention characteristics, and bias-controlled plasticity of this device are fully consistent with the operational requirements of light-stimulated artificial synapses based on 2D semiconductors.

Compared to WSe_2_ neuromorphic devices reported in the literature, which often rely on heterojunctions, the device presented here employs a structurally simple exfoliated WSe_2_ channel, yet exhibits highly stable, long-lived photoinduced conductance states and optically driven plasticity, with paired-pulse facilitation metrics comparable to those of state-of-the-art WSe_2_ neuromorphic devices. Importantly, the plastic state can be efficiently and reversibly switched between long-term and short-term regimes and fully reset only through electrical biasing, enabling robust and reconfigurable long- and short-term synaptic functionality without the need for complex heterostructures or floating-gate integration.

### 3.3 Pavlovian Conditioning and Simulation of an Artificial Neural Network

To further extend the functionalities of the device under study, we performed an experiment based on Pavlovian conditioning.^66^ This simple yet fundamental concept is an example of associative learning. In the biological model, food acts as an unconditioned stimulus. When food is presented, it naturally triggers salivation, which is called the unconditioned response. In contrast, a bell is initially a neutral stimulus because it does not trigger any reaction on its own. However, if the bell is rung at the same time the food is presented, the subject learns to associate the two stimuli.^67^ After this learning process, the neutral stimulus (the bell) becomes linked to the unconditioned stimulus (the food). As a result, the bell alone is sufficient to trigger the salivation.

Experimentally, we mapped the biological inputs to the electrical signals of our device. The gate voltage acts as the food (unconditioned stimulus), while the light acts as the bell (neutral stimulus). We defined a current threshold of 150 nA; any value above this level corresponds to salivation. The food stimulus consists of ten gate spikes at −5 V, and the bell stimulus consists of ten light pulses at 80% power. Both signals have a period of 4 s and the measurement is conducted at a fixed drain bias of 1 V, while the gate voltage is initially grounded. The experimental setup is summarized in Figure 6a. Figure 6b shows the initial state: the neutral stimulus (light) alone generates a current below the 150 nA threshold, meaning no salivation occurs. In Figure 6c, we apply gate voltage pulses (food) alone. This causes the current to exceed 150 nA, triggering salivation. It is important to note that stimulating the device with the gate voltage alone does not enable the light to trigger a response later. This observation confirms that, in the absence of simultaneous pairing, no association is formed, as evidenced by the lack of response during the subsequent light stimulation shown in Figure 6c.

**Figure 6:**
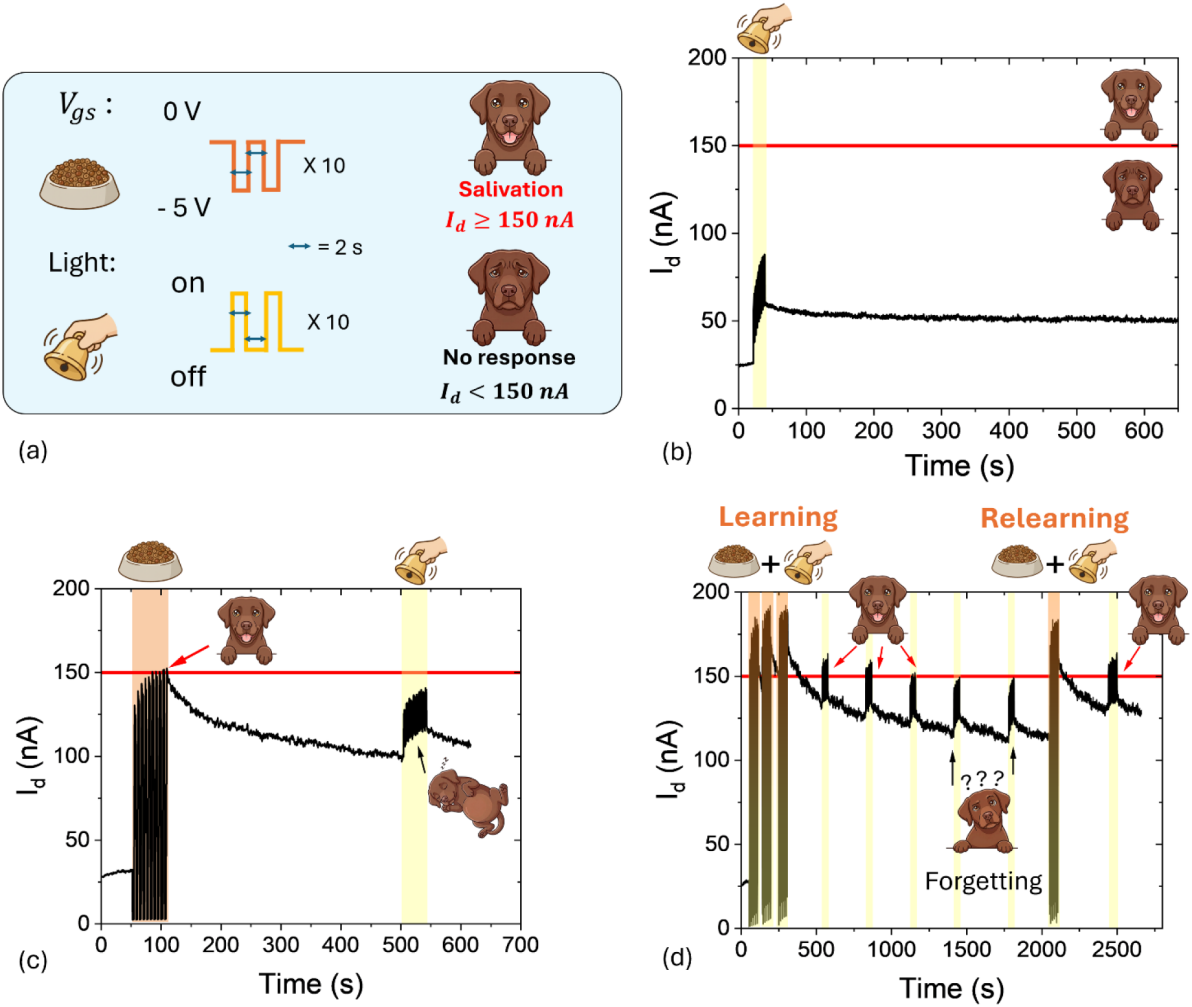
Pavlovian classical conditioning experiment. **a.** Experimental configuration and bias conditions used to mimic the associative learning in Pavlov’s dog. **b.** Response to the neutral stimulus (bell) prior to conditioning. The stimulus alone is insufficient to trigger a significant output signal (salivation). **c.** Control test showing that the unconditioned stimulus (Food) triggers a response, whereas a subsequent isolated bell pulse fails to induce salivation **d.** The simultaneous application of bell and food stimuli (associative learning) strengthens the synaptic weight, allowing the bell alone to trigger the response (salivation). The plot monitors the subsequent decay of the association over time (forgetting) and the successful reestablishment of the connection through a second training.

The learning process is shown in Figure 6d. Here, we applied the light and gate signals simultaneously three times. After this training, the light pulses alone are sufficient to generate a current above 150 nA. This demonstrates that the device has successfully associated the bell with the food. Over time, the device shows a forgetting behavior, but the current remains higher than its initial state, indicating long-term plasticity. A single retraining step is then enough to restore the full response. This behavior is possible because the transfer curve under illumination is hysteretic, allowing repeated pulses to reach and maintain higher current levels.

Pavlovian conditioning is a classical experimental paradigm commonly used to demonstrate the emergence of associative behavior in biological systems. In contrast, current research in neuromorphic engineering primarily designs artificial networks inspired by neural processes. Artificial Neural Networks are computational models consisting of interconnected nodes organized in hierarchical layers.^68^ In these models, input data are processed through weighted connections and activation functions to generate an output, while training is achieved by iteratively adjusting weights via error minimization algorithms such as backpropagation. In this work, image recognition tasks were simulated using NeuroSim V3.0, an open-source framework designed for the implementation of synaptic devices in neuromorphic architectures.^69^ The network architecture was defined as a fully connected Multi-Layer Perceptron comprising three layers: an input layer of 400 neurons, a hidden layer of 100 neurons, and an output layer of 10 neurons, as illustrated in Figure 7a. From a hardware perspective, the physical realization of a large-scale ANN based entirely on 2D materials, such as WSe_2_, remains a formidable challenge at the current state of the art. This difficulty stems from intrinsic technological bottlenecks, including device-to-device variability, challenges in uniform wafer-scale synthesis, and the complex peripheral circuitry required for multiplexed multidevice stimulation and precise conductance control. Consequently, the ANN implemented in this study is entirely simulated in software. This approach serves as a system-level proof of concept, illustrating the potential neuromorphic capabilities of the device while acknowledging the current limits of 2D material integration. The MNIST handwritten digit database was utilized as the data set; specifically, the original images were flattened into 20 × 20 pixel input vectors to match the 400 input neurons. Training was executed using the Stochastic Gradient Descent algorithm over a total of 125 epochs. To optimize computational efficiency and mimic on chip learning constraints, each training epoch utilized a subset of 8000 images randomly selected from the 60000 available training samples, while inference accuracy was evaluated on the full set of 10000 test images. To ensure that the simulation incorporates realistic constraints, key synaptic parameters, specifically the maximum and minimum current (*I*_high_, *I*_low_), read voltage, and pulse duration, were extracted from potentiation characteristics consistent with those employed in the associative learning experiments. To implement the simulation, uniform device performance and linear, symmetric weight update characteristics were assumed across the network. Accordingly, the empirical parameters extracted from a single representative device were universally mapped onto all network nodes.

**Figure 7:**
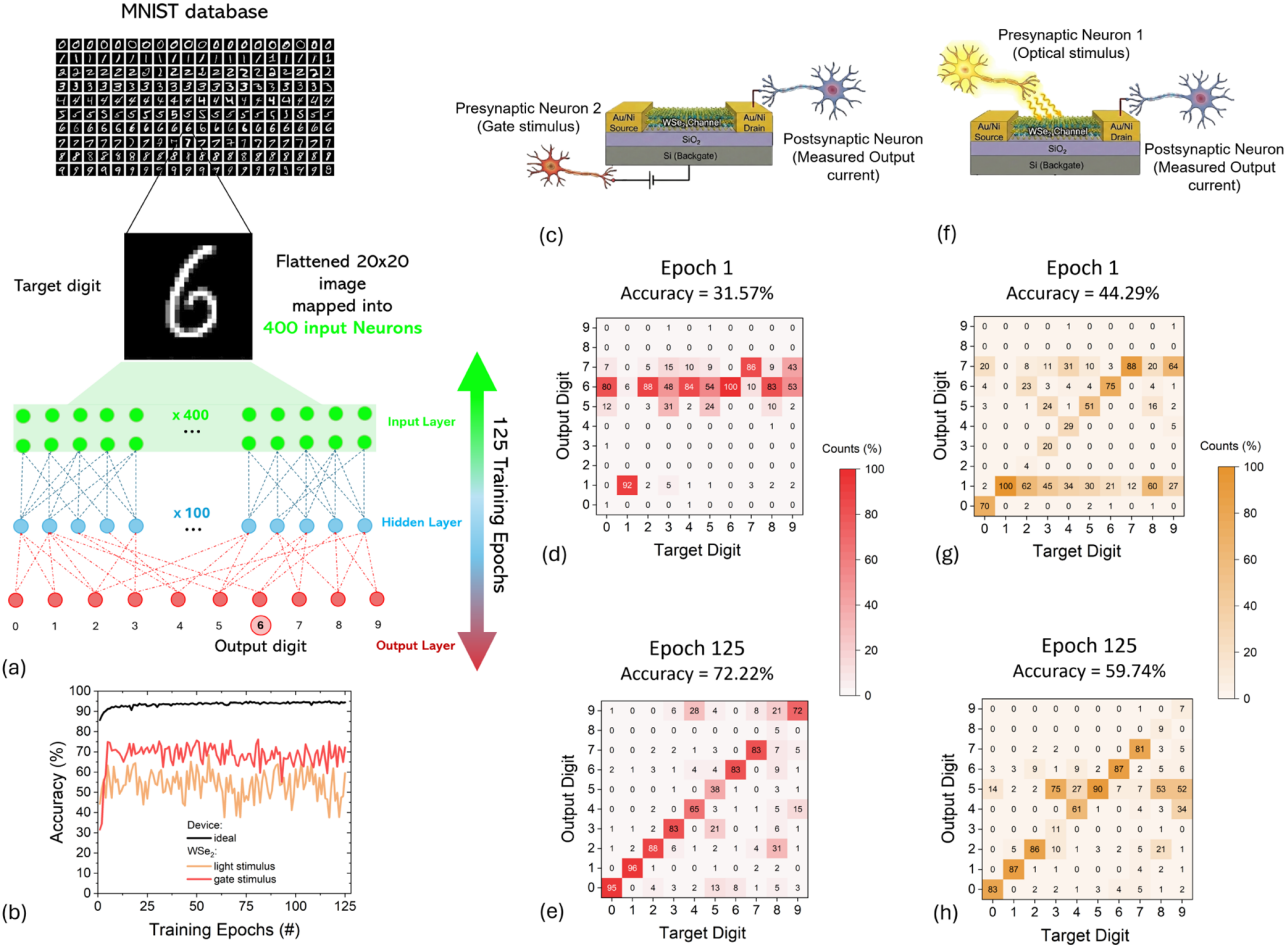
Simulation of an Artificial Neural Network (ANN) for handwritten digit recognition. **a.** Schematic representation of the MNIST digit data set and the layer structure of the simulated ANN. **b.** Comparison of classification accuracy as a function of training epochs for ANNs based on an ideal device (black curve), light-stimulated (orange curve), and gate-stimulated (red curve) WSe_2_ devices. **c-e.** Image recognition simulations using experimental data from gate-stimulated artificial synapses: the device configuration is schematized in (c), while the corresponding confusion matrices show the increase in accuracy from Epoch 1 (d) to Epoch 125 (e). **f-h.** Detailed performance analysis for the light-modulated configuration.

Detailed information regarding the parameter extraction methodology is provided in Figure S5 of the Supporting Information. Specifically, the simulation incorporated physical models for both gate- and light-modulated devices, referred to as gate and optical stimulus configurations. Figure 7b reports the recognition accuracy as a function of training epochs. The performance of the realistic models, both gate-driven (Figure 7c) and light-driven (Figure 7f), is compared with an Ideal Device model, available in the open source of NeuroSim. In addition to classification accuracy, a neural network’s ability to predict all output classes with similar confidence is a very important performance parameter. Confusion matrix is a useful tool for understanding the effectiveness of the neural network model in differentiating the various output classes.^70^ Confusion matrices are arranged as square matrices to compare the predicted output with the ground truth values. The diagonal elements represent the normalized values of number of correct predictions and off-diagonal elements denote the corresponding wrong predictions. The confusion matrices for the gate configuration are reported in Figure 7d-e for Epoch 1 and 125, showing an increment in accuracy until about 72%. The accuracy acquired with the light configuration at epoch 125 is lower (≈60%) because of the lower ratio between the maximum and minimum conductance states, as is clearly visible in Figure S5a-b.

## 4 Conclusions

In this work, we have demonstrated that robust neuromorphic functionality does not necessarily require complex material engineering or multilayered heterostructures. By employing a structurally simple exfoliated WSe_2_ field-effect transistor, we achieved a versatile optoelectronic synapse capable of mimicking essential biological features such as Paired-Pulse Facilitation and associative learning. Our results suggest a different perspective compared to the standard requirement for defect-free devices; rather than eliminating intrinsic nonidealities, we leveraged them to enable synaptic functions. Intrinsic WSe_2_ defects and WSe_2_/oxide interface states are considered the primary trapping centers responsible for the observed persistent photoconductivity. Moreover, trap states at the WSe_2_/oxide interface drive hysteresis in the transfer characteristics, thereby enabling gate-voltage-driven write and erase operations. Crucially, we turned the geometric asymmetry of Schottky contacts, typically considered as a limitation for 2D device performance, into a functional control mechanism. This feature enables the selective switching between Short-Term and Long-Term Plasticity exclusively by tuning the drain bias polarity, allowing for the reconfiguration of synaptic weights without the need for additional floating gates or external memory elements. By validating the device through Pavlovian conditioning experiments and neural network simulations, our findings highlight the potential of WSe_2_ as a platform for energy-efficient and structurally simple brain-inspired systems.

## 5 Experimental Section

Thin WSe_2_ flakes are mechanically exfoliated onto a highly p-doped silicon substrate coated with 85 nm of thermally grown SiO_2_, which serves as the back-gate stack of the field-effect transistor. Metal contacts are then defined by photolithography, followed by electron-beam evaporation of Ni/Au (20 nm/200 nm) and a standard lift-off process.

Raman measurements are performed on the WSe_2_ flakes using a Horiba XploRA Plus Raman microscope equipped with an EMCCD detector and a 532 nm laser excitation.

Electrical characterization was performed in a Lake Shore probe station equipped with gold-coated tungsten tips with a radius of 25 μm. The system allowed measurements under variable temperatures in the range from 77 to 400 K and pressures between 10^–4^ and 10^3^ mbar. Current–voltage characteristics were recorded using a Keithley 4200-SCS parameter analyzer. Optical response experiments were conducted with a SuperK COMPACT supercontinuum laser source (NKT Photonics), delivering white light in the spectral range from 420 to 2400 nm with an available optical power of up to 16.5 mW.

## Supplementary Material



## Notes

Specific electrical characterizations, such as gate leakage current and ambient pressure measurements, an in-depth analysis of the Schottky barrier using the Richardson and Werner-Güttler models, and the estimation of additional parameters related to the neuromorphic behavior of the device. Furthermore, a comparison of the energy consumption per synaptic event with state-of-the-art literature is provided (PDF)
