# Supplementary material for: Mimicking Pavlovian Conditioning with WSe2 Phototransistors
Source: ACS Appl Mater Interfaces. 2026 Jul 3;18(28):39070–82. doi: 10.1021/acsami.6c07765 (PMC13397488; doi:10.1021/acsami.6c07765)
Supplement: Supplementary file 1 [file am6c07765_si_001.pdf]

# Supporting Information

## Mimicking Pavlovian Conditioning with $WSe_2$ Phototransistors

Andrea Sessa[1], Adolfo Mazzotti[1], Kimberly Intonti[1], Aniello Pelella[1], Loredana Viscardi[1], Nadia Martucciello[2], Stephen O'Sullivan[3], Vilas Patil[3], Paul K. Hurley[3,4], Lida Ansari[3], Farzan Gity[3], Antonio Di Bartolomeo[1,*]

[1] Department of Physics "E. R. Caianiello", University of Salerno, via Giovanni Paolo II, Fisciano (SA), 84084, Italy

[2] CNR-SPIN Salerno, via Giovanni Paolo II, Fisciano (SA), 84084, Italy

[3] Tyndall National Institute, University College Cork, Lee Maltings, Dyke Parade, Cork, T12 R5CP, Ireland

[4] School of Chemistry, University College Cork, Cork, T12 R5CP, Ireland

[*]Corresponding author's e-mail: adibartolomeo@unisa.it

### 1. Gate Leakage Current Control

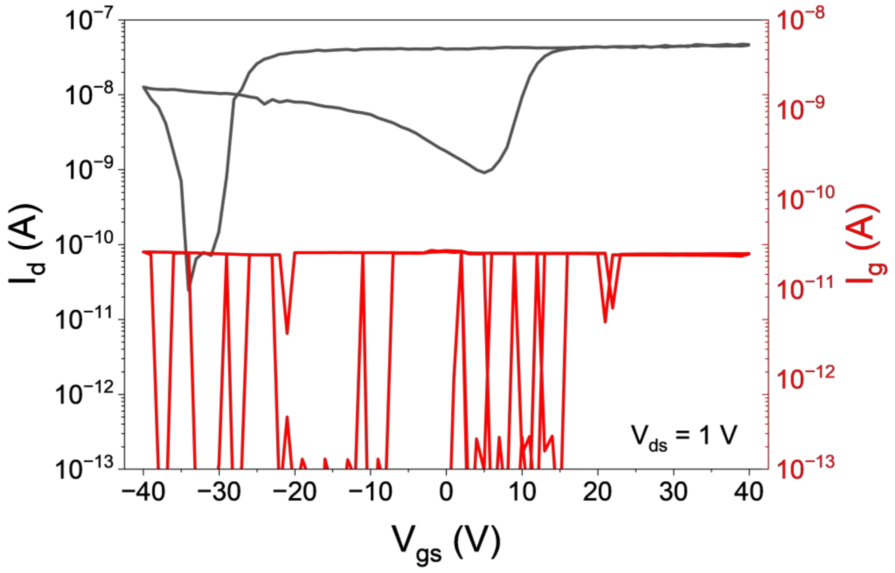


**Figure S1.** Gate leakage current recorded during the transfer curve measurement at room temperature with Vds = 1 V. The negligible magnitude of the gate current compared to the drain current confirms the integrity of the dielectric oxide and the absence of significant gate leakage.

### 2. Preliminary characterization in air

Before the high-vacuum characterization, the device was tested under ambient temperature and pressure, as summarized in Figure S2. The transistor exhibits significant Schottky contact asymmetry, potentially related to air-induced barrier inhomogeneities[1], alongside a robust photoresponse. The transfer characteristics display pronounced hysteresis, likely related to the interaction of ambient adsorbates with the $WSe_2$ flake[2,3]. Furthermore, as shown in Figure S2(c),

the device successfully processes a train of 150 optical pulses ($t_{on}$ = 50 ms, $t_{off}$ = 200 ms), demonstrating stable synaptic behavior even under ambient conditions.

The experimental characterization was systematically conducted under vacuum conditions to decouple the extrinsic effects of atmospheric adsorbates from the intrinsic features of both the material and the device architecture. This isolation is particularly critical during concurrent optical stimulation and electrical gate pulsing. Consequently, the vacuum environment established a clearer and more unambiguous physical interpretation of the device's underlying opto-neuromorphic mechanisms.

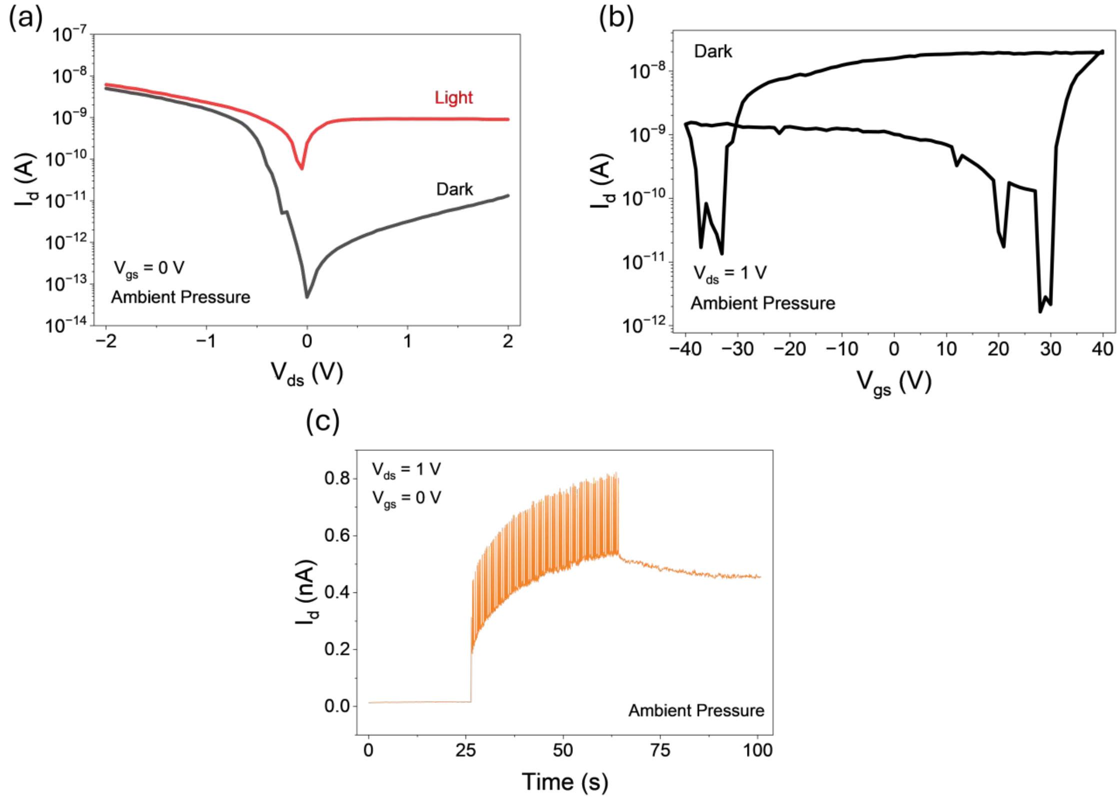


**Figure S2.** Preliminary characterization in ambient pressure and at room temperature of the $WSe_2$-based FET. (a) IV curve at grounded gate both in the dark and under illumination (white laser, 80% power). (b) Transfer curve at $V_{ds}$ = 1 V in the dark. (c) Response of the device to illumination with a train of 150 pulses with $t_{on}$ = 50 ms, $t_{off}$ = 200 ms.

## 3. Schottky barrier characterization and Werner-Güttler model for the barrier inhomogeneity

To determine whether the observed IV asymmetry stems exclusively from the disparity in contact area (*S*) rather than differences in barrier height or inhomogeneity-related effects, we evaluated the Schottky barrier height at both interfaces. Temperature-dependent IV characteristics were

recorded from 300 to 380 K at zero gate bias and are reported in Figure S3a. The reverse saturation current $I_0$ was extracted for each temperature from the y-intercept of the linear fit to the semi-logarithmic IV curves in the thermionic emission regime. The barrier height was subsequently determined using the following expression[4]:

$$\ln\left(\frac{I_0}{T^2}\right) = \ln(SA^*) - \frac{q\Phi_B}{kT} \ (S1)$$

The Richardson plot analysis enables the simultaneous extraction of the Schottky barrier height ( $q\Phi_B$) and the Richardson constant ($A^*$) for both interfaces (Figure S3b). In our measurement setup, the bias voltage is applied to Contact A, as depicted in Figure 1(b). Consequently, under a positive $V_{ds}$, electrons must overcome the Schottky barrier at Contact B, whereas under a negative $V_{ds}$, electron injection is limited by the Schottky barrier at Contact A. Specifically, nearly identical barrier heights of $0.48 \pm 0.02\ eV$ for the contact A and $0.53 \pm 0.03\ eV$ for the contact B were obtained.

To verify that barrier inhomogeneities do not significantly affect the evaluation of $q\Phi_B$, we further characterized the contact interfaces using the Werner-Güttler model[5]. Within this framework, the effective barrier height is described by the following expression:

$$q\Phi_B^{eff} = kT \ln\left(\frac{SA^*T^2}{I_0}\right)(S2)$$

To account for potential inhomogeneities at the metal-semiconductor interface, this model assumes that the Schottky barrier height is not perfectly uniform but instead follows a Gaussian distribution characterized by a mean value $q\Phi_0$ and a standard deviation $q\sigma$. The effective barrier height $q\Phi_B^{eff}$ is expressed as follows

$$q\Phi^{eff}{}_B = q\Phi_0 - \frac{(q\sigma)^2}{2kT}(S3)$$

By performing a linear fit of the extracted effective barrier heights against 1/(2kT), we determined both the mean Schottky barrier height and the corresponding standard deviation for each contact (Figure S3c). This analysis revealed a standard deviation of approximately 0.01 eV for both interfaces. Such a negligible value falls within the experimental uncertainty associated with the Richardson method. Consequently, these findings demonstrate that the device's electrical behavior is not significantly influenced by barrier fluctuations. This further validates the conclusion that the observed asymmetry in the I-V characteristics is fundamentally rooted in the geometric disparity of the contact areas rather than interfacial non-idealities.

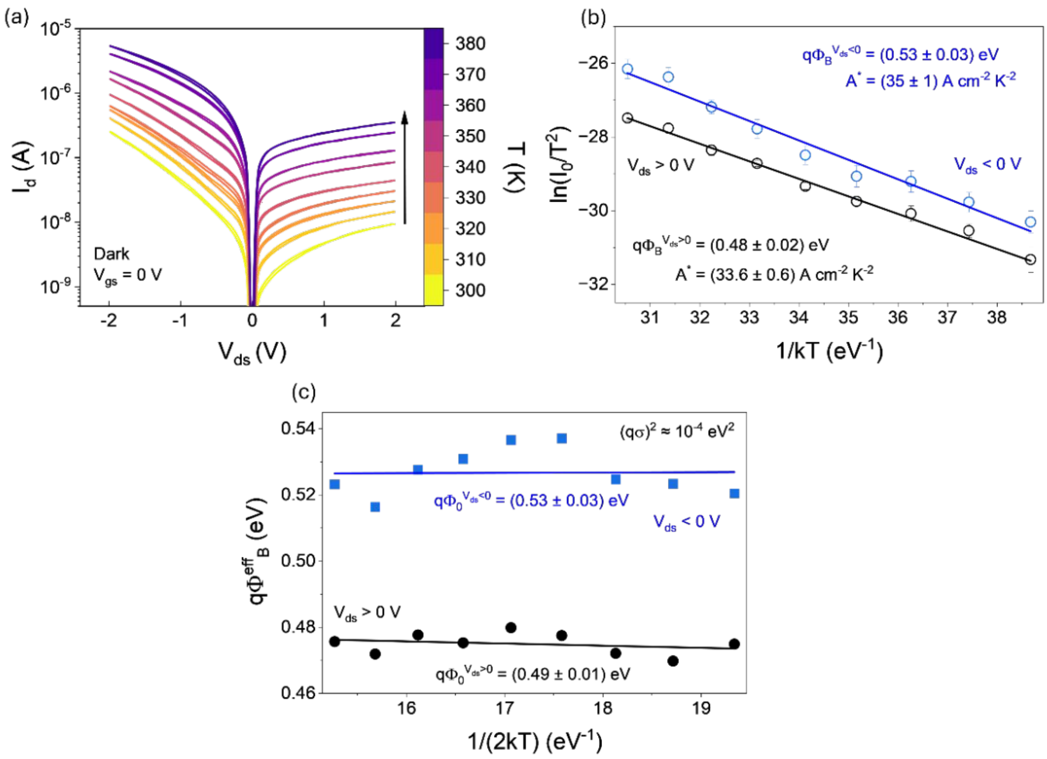


**Figure S3.** Schottky barrier characterization via Richardson and Werner-Güttler analyses. (a) Temperature-dependent IV characteristics recorded at zero gate bias from 300 K to 380 K in 10 K increments. (b) Richardson plots extracted for the two contact interfaces to determine the barrier height and Richardson constant. (c) Werner-Güttler analysis of the effective barrier height, demonstrating negligible Schottky barrier inhomogeneities for both contacts.

## 4. Parameters characterization of the synaptic behavior of the $WSe_2$-based FET

Figure S4 illustrates the temporal evolution and double-exponential fitting of the photocurrent decay following a ten-pulse optical train at $V_{ds} = -1\ V$ (blue curve) and $V_{ds} = 1\ V$ (purple curve). The double-exponential decay is modeled using the following expression:

$$I_{ph} = C_0 + C_1 \exp\left(-\frac{\Delta t}{\tau_1}\right) + C_2 \exp\left(-\frac{\Delta t}{\tau_2}\right) (S4)$$

Where, $\tau_1$ and $\tau_2$ represent the fast and slow characteristic time constants of the recombination process, respectively. The accelerated decay kinetics observed at $V_{ds} = -1\ V$ compared to the positive bias confirm the transition from long-term to short-term memory functionality upon reversing the drain bias polarity.

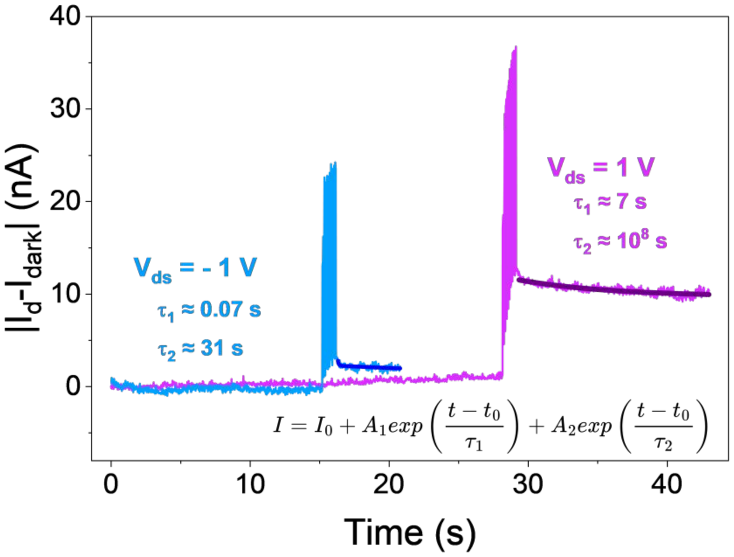


**Figure S4.** Temporal evolution and double-exponential fitting of the photocurrent decay following a ten-pulse optical train at $V_{ds} = -1\ V$ (blue) and $V_{ds} = 1\ V$ (purple).

The parameters for the ANN simulation were derived by fitting the photocurrent decay with an exponential function following the cessation of both gate (Figure S5(a)) and optical (Figure S5(b)) stimuli. Specifically, the high-state current levels were extracted from the exponential saturation values; this approach ensures the use of a stable steady-state current rather than the transient peaks observed immediately after stimulation. Read operations were conducted at a drain bias of $V_{ds} = 1\ V$ with a grounded gate. Similarly, the baseline level corresponds to the dark current measured at $V_{ds} = 1\ V$ under the same grounded gate condition.

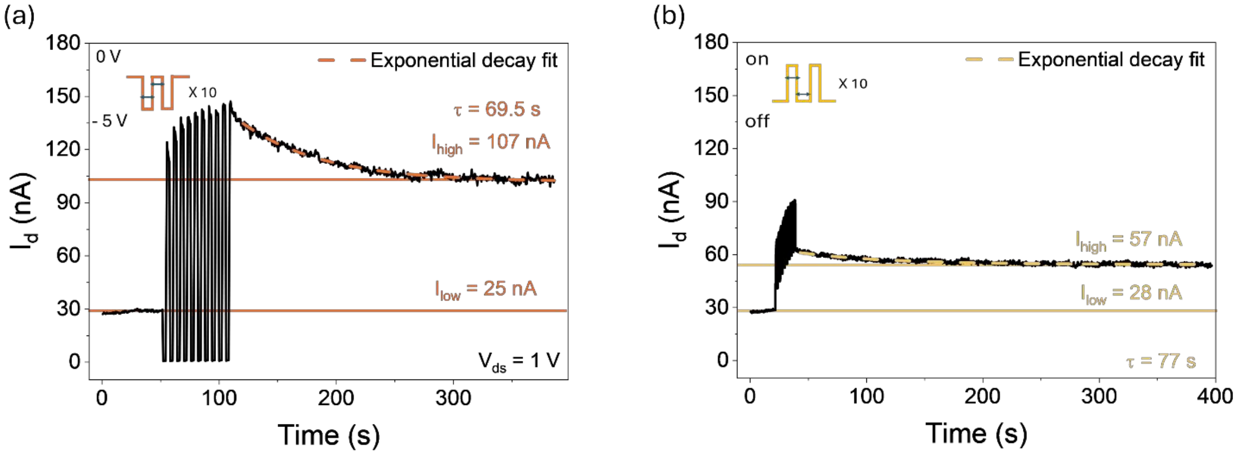


**Figure S5.** Parameters extraction for the simulation of the ANN for both the gate (a) and optical (b) stimulus.

## 5. Energy consumption per individual synaptic event and comparison with literature

To evaluate the energy efficiency of our device, we estimated the energy consumption for individual synaptic events based on our experimental parameters. For an optical potentiation event, the energy is calculated as[6]

$$E_{opt} = P_{inc} \cdot t_{on} \quad (S5)$$

Utilizing the parameters from the dynamic measurements the estimated optical energy consumption is ~22 nJ per spike. Similarly, the electrical energy for a gate depression event is calculated by accounting for the power dissipated in the channel and the capacitive charging of the gate oxide, yielding an estimation of ~150 nJ per pulse. Table S1 compares these metrics with other state-of-the-art light-stimulated 2D materials-based neuromorphic devices. It is crucial to note that the energy values reported for our device represent a macroscopic upper bound also dictated by the limitations of the measurement equipment.

| Material | Architecture | Working Principle | Energy or Power Consumption | Ref. |
|---|---|---|---|---|
| $MoS_2$ monolayer | Three-Terminal device | $MoS_2$ monolayer arrays assisted by gold nanorods | 77.6 pJ | [7] |
| $WS_2$ | Three-Terminal Device | Heterosynaptic memtransistor | 150 aJ | [8] |
| $MoSe_2$ | Two-terminal device | Multilayer $MoSe_2$ moiré superlattice | 10 fJ | [9] |
| $WSe_2$ Quantum Dot | Two-terminal device | Memristor | 0.16 nW | [10] |
| $WSe_2$ | Three-Terminal Device | Lewis Acid Doping | 0.1 fJ | [11] |
| Gr/$WSe_2$ | Three-Terminal Device | Van der Waals Heterostructure | 127 aJ | [12] |

| | | | | |
|---|---|---|---|---|
| $WSe_2$ | Three-terminal device | Asymmetrical Schottky contacts | 22 nJ | **This work** |

**Table S1.** Comparison of energy consumption among state-of-the-art 2D materials-based artificial synapses.

## References


(1) Sessa, A.; De Stefano, S.; Durante, O.; Pelella, A.; Aldrigo, M.; Parvulescu, C.; Dinescu, A.; Kuo, C.-N.; Lue, C. S.; Dadiani, T.; D'Olimpio, G.; Faella, E.; Politano, A.; Passacantando, M.; Di Bartolomeo, A. Synaptic Behavior in SnSe2 Field-Effect Transistors Induced by Surface Oxide and Trap Dynamics. *Adv. Electron. Mater.* **2026**, *12* (5), e00734. https://doi.org/10.1002/aelm.202500734.

(2) Di Bartolomeo, A.; Kumar, A.; Durante, O.; Sessa, A.; Faella, E.; Viscardi, L.; Intonti, K.; Giubileo, F.; Martucciello, N.; Romano, P.; Sleziona, S.; Schleberger, M. Temperature-Dependent Photoconductivity in Two-Dimensional MoS2 Transistors. *Mater. Today Nano* **2023**, *24*, 100382. https://doi.org/10.1016/j.mtnano.2023.100382.

(3) Mazzotti, A.; Intonti, K.; Sessa, A.; Viscardi, L.; Durante, O.; Pelella, A.; O'sullivan, S.; Patil, V.; Hurley, P.; Ansari, L.; Gity, F.; Bartolomeo, A. D. Defect-Tuned Conduction in Ultrathin MoTe2 Field-Effect Transistors. *J. Mater. Chem. C* **2026**. https://doi.org/10.1039/D6TC00385K.

(4) Di Bartolomeo, A.; Intonti, K.; Peluso, L.; Di Marco, R.; Vocca, G.; Romeo, F.; Giubileo, F.; Grillo, A.; Orhan, E. Metal-Semiconductor Schottky Diode with Landauer's Formalism. *Nano Express* **2025**, *6* (2), 022501. https://doi.org/10.1088/2632-959X/ade460.

(5) Werner, J. H.; Güttler, H. H. Barrier Inhomogeneities at Schottky Contacts. *J. Appl. Phys.* **1991**, *69* (3), 1522–1533. https://doi.org/10.1063/1.347243.

(6) Wen, F.; Meng, Y.; Zhou, H.; Chen, X. Optoelectronic Synapse Based on a BiI3/Bi2Se3 van Der Waals Heterostructure for Neuromorphic Visions. *Appl. Phys. Lett.* **2026**, *128* (10), 103301. https://doi.org/10.1063/5.0316774.

(7) Huang, M.; Ali, W.; Yang, L.; Huang, J.; Yao, C.; Xie, Y.; Sun, R.; Zhu, C.; Tan, Y.; Liu, X.; Li, S.; Li, Z.; Pan, A. Multifunctional Optoelectronic Synapses Based on Arrayed MoS2 Monolayers Emulating Human Association Memory. *Adv. Sci.* **2023**, *10* (16), 2300120. https://doi.org/10.1002/advs.202300120.

(8) Liu, Y.; Cai, D.; Zhao, T.; Shen, M.; Liu, X.; Gu, D. Heterogeneous Ion-Modulated 2D-WS2 Heterosynaptic Memtransistor for Controllable Synaptic Modulation and Energy-Efficient Neuromorphic Computing. *Chem. Eng. J.* **2024**, *492*, 152215. https://doi.org/10.1016/j.cej.2024.152215.

(9) Yang, H.; Hu, Y.; Zhang, X.; Ding, Y.; Wang, S.; Su, Z.; Shuai, Y.; Hu, P. Near-Infrared Optical Synapses Based on Multilayer MoSe2 Moiré Superlattice for Artificial Retina. *Adv. Funct. Mater.* **2024**, *34* (2), 2308149. https://doi.org/10.1002/adfm.202308149.

(10) Wang, Z.; Wang, W.; Liu, P.; Liu, G.; Li, J.; Zhao, J.; Zhou, Z.; Wang, J.; Pei, Y.; Zhao, Z.; Li, J.; Wang, L.; Jian, Z.; Wang, Y.; Guo, J.; Yan, X. Superlow Power Consumption Artificial Synapses Based on WSe2 Quantum Dots Memristor for Neuromorphic Computing. *Research* **2022**, *2022*, 9754876. https://doi.org/10.34133/2022/9754876.

(11) Ma, M.; Huang, C.; Yang, M.; He, D.; Pei, Y.; Kang, Y.; Li, W.; Lei, C.; Xiao, X. Ultra-Low Power Consumption Artificial Photoelectric Synapses Based on Lewis Acid Doped WSe2 for Neuromorphic Computing. *Small* **2024**, *20* (51), e2406402. https://doi.org/10.1002/smll.202406402.

(12) Tang, H.; Anwar, T.; Jang, M. S.; Tagliabue, G. Light-Intensity Switching of Graphene/WSe2 Synaptic Devices. *Adv. Sci.* **2024**, *11* (24), 2309876. https://doi.org/10.1002/advs.202309876.
